# The effect of ultrasound-guided single right stellate ganglion block on postoperative first night sleep in maxillofacial surgery patients: a single center randomized controlled study

**DOI:** 10.1097/MS9.0000000000003732

**Published:** 2025-08-21

**Authors:** Ye Wang, Lei Wang, Zibin Jin, Chao Wen, Lingxin Wei, Dong Yang, Xiaoming Deng

**Affiliations:** Department of Anesthesiology, Plastic Surgery Hospital, Chinese Academy of Medical Sciences and Peking Union Medical College, Beijing, China

**Keywords:** maxillofacial surgery, postoperative sleep disorders, Richard–Campbell Sleep Questionnaire (RCSQ), stellate ganglion block

## Abstract

**Purpose:**

To observe the effect of ultrasound-guided single right stellate ganglion block (SGB) on postoperative first night sleep in patients undergoing maxillofacial surgery.

**Methods:**

This study included 120 participants who underwent maxillofacial surgery under general anesthesia from 11 April 2023 to 31 August 2024. These participants were divided into three groups, namely blank control group, 1% lidocaine group, and 2% lidocaine group, with 40 patients in each group. Their blood pressure, heart rate, blood oxygen saturation, and the visual analog scale (VAS) scores were recorded before the operation (baseline), immediately after awakening, immediately after arrival at the anesthesia intensive care unit (AICU), 1 h after arrival at the AICU, and 2 h after arrival at the AICU. The Richard–Campbell Sleep Questionnaire (RCSQ) was used to evaluate the sleep quality in the three patient groups on the first night after surgery. Complications associated with SGB and the number of sedative analgesic medication doses supplemented on the first postoperative night were recorded.

**Results:**

The RCSQ scores of the blank control group, 1% lidocaine group, and 2% lidocaine group were 44.5 (33.3, 62.0), 67.0 (54.0, 87.9), and 96.0 (65.8, 100.0), respectively, and the scores gradually increased (comparison among three groups: *P* < 0.0001). There was a statistical difference among the three groups in the gradual slowing down of heart rate after extubation and wakefulness, immediately after arrival at the AICU, 1 h after arrival at the AICU, and 2 h after arrival at the AICU. There were no significant complications of SGB in the three groups. The 1% and 2% lidocaine group reduced the number of postoperative sufentanil remedies.

**Conclusion:**

Ultrasound-guided single right SGB effectively improved the quality of postoperative first night sleep according to RCSQ and reduced stress reactions within 2 h in patients undergoing maxillofacial surgery. Notably, the efficacy of 4 mL of 2% lidocaine was superior to that of 4 mL of 1% lidocaine, making it a safe and effective method for improving postoperative sleep.

## Introduction

Maxillofacial surgery involves changing a patient’s skeletal structure to make the facial appearance normal or achieve an aesthetically favorable outcome. The intraoperative osteotomy procedure involves significant trauma, blood loss, and postoperative discomfort, which can significantly impact patients’ postoperative sleep and recovery quality^[1,2]^. Comfort management during the perioperative period is particularly important in pursuing aesthetic results after plastic surgery, and optimizing sleep management for maxillofacial surgery patients is crucial^[3,4]^. Clinical studies have confirmed that stellate ganglion block (SGB) can effectively alleviate sleep disorders and insomnia. However, due to individualized anatomical differences and inaccurate positioning of the nerve block during blind exploration, complications like nerve block failure and peripheral vascular and nerve damage may occur. Such safety issues limit its widespread application in the clinical treatment of sleep disorders^[5,6]^. Ultrasound is a valuable tool for imaging soft tissue and bone surfaces, and its use allows for the advancement of the puncture needle to be guided under direct vision for precise positioning. Furthermore, it offers the ability to observe the spread of the injected fluid at the target site. Moreover, it reduces the dose of injected medication and minimizes the risk of radiographic exposure to both medical practitioners and patients^[7]^. Unlike left-sided SGB, right-sided SGB has a stabilizing effect on myocardial function^[8]^. Although ultrasound technology has been widely used for SGB, few studies have explored the effects of ultrasound-guided right-sided SGB on postoperative sleep in maxillofacial surgery patients.

**HIGHLIGHTS**

- Use non-pharmacological methods to intervene in postoperative first night sleep in patients undergoing maxillofacial surgery.
- Use ultrasound guidance for nerve block to reduce patient damage.
- Use randomized controlled trials for comparison.

This study observed the effect of ultrasound-guided administration of two different concentrations of lidocaine for single right SGB on postoperative first night sleep in patients undergoing maxillofacial surgery. Neither the research process nor the completion of the manuscript utilized artificial intelligence technology. This was clarified in the TITAN Guideline Checklist 2025^[9]^.

### Research hypothesis

This study included a total of 120 patients undergoing maxillofacial surgery. All participants were randomly divided into three groups: a blank control group (BC group, n = 40), a 1% lidocaine group (1% Lido group, n = 40), and a 2% lidocaine group (2% Lido group, n = 40). Ultrasound-guided right SGB can safely and effectively alleviate sleep disorders experienced by patients on the first night after undergoing maxillofacial surgery.

## Methods

### Study participants

This study was a single-center, interventional, double-blind randomized controlled study. The protocol was approved by the Hospital Ethics Committee (Z202351), and the study was registered with the China Clinical Trials Registration Centre (http://www.chictr.org.cn) before participant recruitment (date of registration: 10 April 2023). All participants were informed of the purpose of this study and provided signed informed consent. This study, from participant inclusion to research design, implementation, data collection, and disclosure, was entirely in compliance with the Declaration of Helsinki.

We enrolled 120 patients (20 men, 100 women; age, 18–40 years) with ASA class I or II who underwent maxillofacial surgery under general anesthesia between 11 April 2023 and 31 August 2024. Given that we comprehensively informed the patients about the efficacy of SGB before the operation, the patient participation was high and no cases were excluded. Furthermore, the procedure was painless as the patients were still under anesthesia when SGB was performed before removing the tracheal tube after the surgery. Furthermore, the patients were also willing to answer follow-up questions after the operation. The first night of postoperative stay was in the anesthesia intensive care unit (AICU), and all patients chose not to use self-controlled analgesic pumps in the postoperative period. Patients with allergy to lidocaine, preoperative sleep disorders requiring them to take sleep-promoting drugs, and other systemic diseases (e.g., congenital heart disease, hypertension, and epilepsy) were excluded. Patients with neck foreign bodies, coagulation disorders, cervical spondylosis, severe sleep apnea syndrome, respiratory foreign bodies, tumors, polyps, abscesses in the upper respiratory tract, and upper respiratory tract infections in the last 2 weeks were excluded. Furthermore, patients with hearing and speech disorders that prevented them from co-operating in the study and those who refused enrollment in the study were excluded.

### Random and blind methods

**Figure 1.**
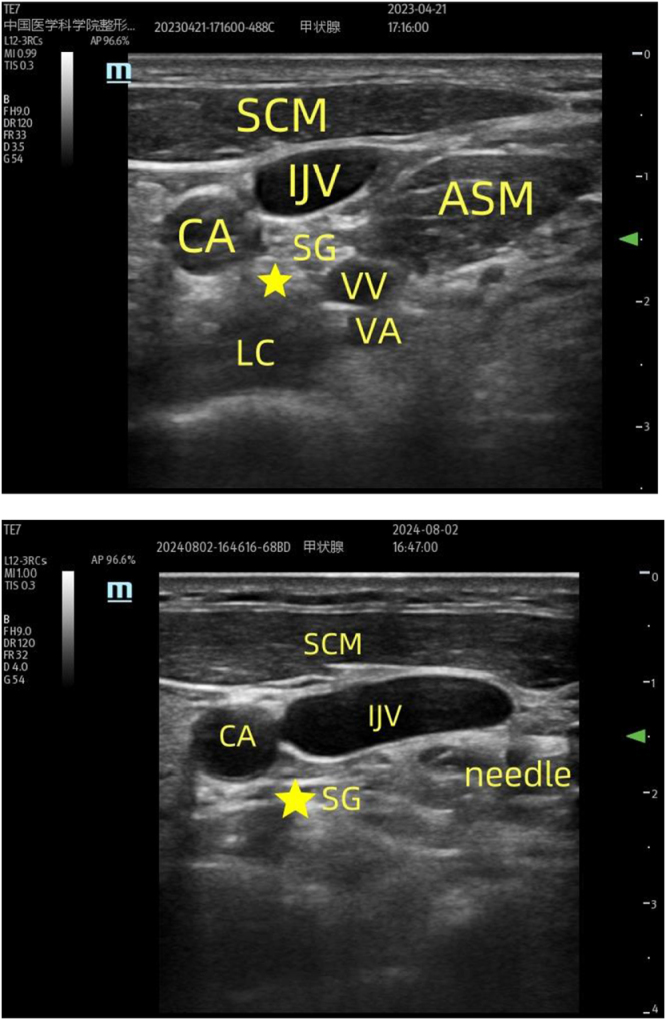
Ultrasound-guided stellate ganglion block. SG, stellate ganglion; SCM, sternocleidomastoid; ASM, anterior scalenus muscle; CA, carotid artery; IJV, internal jugular vein; VV, vertebral vein; VA, vertebral artery; LC, longus colli.

This study included a total of 120 patients undergoing maxillofacial surgery. All participants were randomly divided into three groups using a simple computer randomization method and a sealed opaque envelope method in a 1:1:1 ratio: a blank control group (BC group, n = 40), a 1% lidocaine group (1% Lido group, n = 40), and a 2% lidocaine group (2% Lido group, n = 40). Ultrasound-guided right SGB (Fig. 1) was initiated before the removal of the nasotracheal tube by an anesthesiologist with over 5 years of experience. Follow up was performed by another anesthesiologist who was blinded to the patient’s grouping, and the patient was blinded to the grouping as well. Data analysis was performed by another anesthesiologist.

### Anesthesia procedure

The patients were routinely fasted from food and water for 8 h, and electrocardiogram (ECG), oxygen saturation (SpO_2_), heart rate (HR), and blood pressure (BP) were monitored after admission. After establishing intravenous access, 0.05-mg/kg midazolam and 0.2-μg/kg sufentanil were administered.

Once the patient was sedated, mask ventilation was started, and a cotton swab moistened with ephedra 10 mg was placed in selected nostrils. Thereafter, 2.0-mg/kg propofol and 0.2-mg/kg cis-atracurium were intravenously injected, followed by continuous mask ventilation for 2 min. Nasotracheal intubation (Polar Preformed Tracheal Tube, Smith Medical International Limited, UK) was started after the mandible was relaxed. Then, the patients were connected to the anesthesia machine for intermittent barotropic ventilation. Anesthesia was maintained with 7 mg · kg^−1^h^−1^ propofol or 1–2% sevoflurane and 2 µg · kg^−1^min^−1^ remifentanil, with a tidal volume (VT) of 8–10 mL/kg, respiratory rate of 12–15 breaths/min, flow rate of 2.5 L/min, and the O_2_: air ratio of 1.0:1.5 L/min. Intraoperative control of BP was ensured to reduce blood loss.

### Surgical classification

Single surgery was defined as undergoing mandibular angle and masseter resection, maxillary Lefort I osteotomy, or mandibular sagittal split osteotomy. Multiple surgeries were defined as undergoing two or more of the following procedures: maxillary Lefort I osteotomy, mandibular sagittal split osteotomy, mandibular angle/zygomatic/chin osteotomy, and masseter resection.

### Procedures

**Figure 2.**
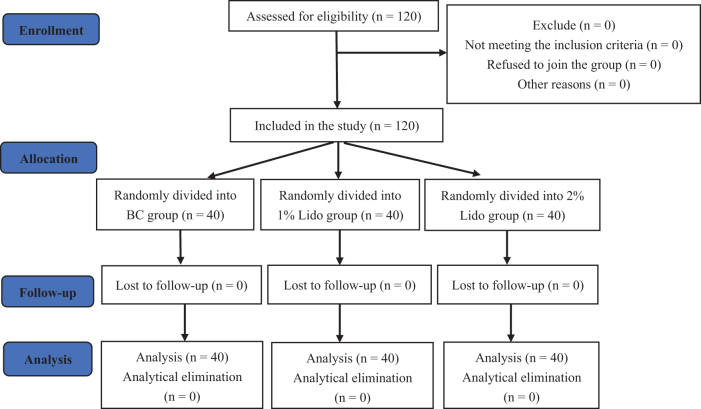
Flow chart.

Using a computer, 120 patients were randomized into three groups (Fig. 2), namely a blank control group (n = 40), in which no intervention was given, 1% lidocaine group (n = 40), and 2% lidocaine group (n = 40). In the latter two groups, 4 mL of 1% or 2% lidocaine was respectively used to perform a right SGB under ultrasound guidance for the treatment of postoperative sleep disturbances. This administration was performed before the end of the operation and removal of the endotracheal tube. After removing the nasotracheal intubation and after the patient regained consciousness and could respond clearly according to instructions, we evaluated whether the SGB was successful. The presence of Horner’s syndrome indicated successful nerve block.

The blood pressure, heart rate, blood oxygen saturation, and the visual analog scale (VAS) scores of the three patient groups were recorded before surgery at baseline, immediately upon awakening, immediately after returning to the AICU, 1 h after returning to the AICU, and 2 h after returning to the AICU. In the night, if the VAS pain score was 4–6 points, the patient was given oral oxycodone and acetaminophen tablets (5 mg per adult per session). If the VAS pain score was 7–10 points, intravenous 0.5-μg/kg sufentanil was administered. If the patient could still not fall asleep after 0:00, 0.02 mg/kg midazolam was intravenously administered. In the event of respiratory depression, the patient was woken up immediately and given oxygen with mask ventilation. In the AICU, the patients continuously receive oxygen inhalation through a Venturi mask, and one anesthesiologist on duty and two nurses on duty continuously monitor the patients.

On the second day, the Richard–Campbell Sleep Questionnaire (RCSQ) was used to evaluate the sleep quality of the three patient groups on the first night after surgery when the patients were completely awake^[10]^.

The RCSQ was a subjective assessment tool designed to evaluate sleep quality in hospitalized patients, particularly those in intensive care unit (ICU). Developed by Richard and Campbell in 1987, it was a simple, quick-to-administer instrument widely used for preliminary screening of sleep disturbances in clinical settings. The strengths of the RCSQ were: quick administration (2–5 minutes), ideal for critically ill patients with limited stamina; tailored to hospital environments; validated for reliability and sensitivity, translated into multiple languages (e.g., Chinese, Spanish).

The scale had questions evaluating five aspects: RCSQ 1. Depth of sleep last night, RCSQ 2. Speed of falling asleep last night, RCSQ 3. Number of awakenings last night, RCSQ 4. Speed of falling asleep again after waking up last night, and RCSQ 5. Quality of sleep last night, with scores ranging from 0–100 points per question. A higher score indicated a better situation. The RCSQ score was the average score of five items, categorized as: >70 good sleep quality, 50–70 moderate sleep quality, <50 poor sleep quality^[10]^.

The complications occurring as a result of the SGB, such as bleeding, arm numbness, and recurrent laryngeal nerve block, were recorded, and a record of postoperative sedative and analgesic drugs supplemented was maintained as well. We compared the effects of ultrasound-guided single right SGB for two different lidocaine concentrations on postoperative first night sleep in patients undergoing maxillofacial surgery.

### Primary parameters

RCSQ1, RCSQ2, RCSQ3, RCSQ4, RCSQ5, and the RCSQ score (average score of the RCSQ 1–5).

### Secondary parameters

The changes in blood pressure, heart rate, blood oxygen saturation, and VAS scores in the three patient groups were recorded at the aforementioned time points. The complications occurring as a result of the SGB were recorded, and a record of postoperative sedative and analgesic drugs supplemented was maintained as well.

### Sample size calculation

First, a pre-trial treatment was conducted to observe the effect of right SGB on the sleep quality of the patients on the first night after surgery. The total sample size was calculated on the basis of our previous study that compared the effects of different concentrations of dexmedetomidine nasal spray on postoperative sleep of maxillofacial surgery patients in the AICU^[11]^. Compared with patients in the blank control group (n = 39) and 1.0-µg/kg dexmedetomidine group (n = 39), those in the 1.5-µg/kg dexmedetomidine group (n = 39) had significantly prolonged N3 sleep time, lower incidence of postoperative sore throat, and shorter average hospital stay (*P* < 0.05)^[11]^. We assume that SGB can statistically significantly improve postoperative sleep in maxillofacial surgery patients. Based on α = 0.05, 1-β = 0.8, and a data loss rate of 10%, the Power Analysis and Sample Size software (version 11.0; NCSS, Kaysville, Utah, United States) was used to calculate the sample size, with 40 cases in each group and a total of 120 cases.

### Statistical analysis

All statistical analyses were performed using the Statistical Package for Social Sciences software (version 26.0; SPSS Inc., Chicago, IL, United States). For the three patient groups, demographic features, namely age, weight, height, and BMI, were presented as means ± standard deviations and ranges, whereas sex, the ASA class, and surgical complexity were presented as percentages. Intraoperative drug use and RCSQ scores were expressed as medians. Hemodynamic changes were expressed as mean ± standard deviation. The number of postoperative adverse events and sedative–analgesic drug refills were expressed as frequencies and percentages. The Shapiro–Wilk test was used to analyze whether the data were normally distributed. For normally distributed data, the analysis of variance (*ANOVA*) was used to compare differences among the three groups. The least significant difference (*LSD*) test was used for *post-hoc* pairwise comparisons. The Kruskal–Wallis H test was used to compare non-normally distributed data among the three groups. The chi-square test was used to compare proportions. The *P* value of <0.05 was considered statistically significant.

## Results

There were no statistically significant differences in age, height, weight, Body Mass Index (BMI), sex ratio, the ASA scale, and surgical complexity among the three patient groups (as shown in Table 1). There was also no statistically significant difference in intraoperative drug use among the three patient groups (as shown in Table 2).

**Table 1:** Participant demographics and surgical complexity

Variables	BC group (n = 40)	1% Lido group (n = 40)	2% Lido group (n = 40)	*F*-value	Comparison among three groups, *P* value
Age (years) ( $\overset{ˉ}{x}\pm s$, range)	29.3 ± 6.1 [19–40]	27.3 ± 5.5 [18–40]	28.3 ± 5.2 [18–40]	1.311	0.274
Weight (kg) ( $\overset{ˉ}{x}\pm s$, range)	54.4 ± 7.6 [42.5–73]	55.4 ± 9.3 [43.0–91.5]	56.3 ± 9.3 [40.0–87.0]	0.452	0.638
Height (cm) ( $\overset{ˉ}{x}\pm s$, range)	165.6 ± 5.9 [157.0–180.0]	168.0 ± 8.5 [151.0–186.0]	164.1 ± 6.1 [153.0–180.0]	3.072	0.050
BMI (kg/m^2^) ( $\overset{ˉ}{x}\pm s$, range)	19.8 ± 2.3 [17.0–24.8]	19.6 ± 2.4 [14.9–27.3]	20.8 ± 2.7 [16.4–30.1]	2.729	0.069
Sex (case, %)				χ ^2^-value	*P* value
Male	6 (5)	10 (25)	4 (10)	3.360	0.186
Female	34 (85)	30 (75)	36 (90)		
ASA class (case, %)					
ASA I	36 (90.0)	35 (87.5)	33 (82.5)	1.010	0.604
ASA II	4 (10.0)	5 (12.5)	7 (17.5)		
Surgical complexity (case, %)					
Single	13 (32.5)	9 (22.5)	8 (20.0)	1.867	0.393
Multiple	27 (67.5)	31 (77.5)	32 (80.0)		

*P* <0.05 indicates a statistically significant difference. ANOVA was used to compare age, height, weight, and BMI, and the *χ ^2^* test was used to compare sex, ASA class, and surgical complexity.

**Table 2:** Comparison of intraoperative conditions of three groups of participants M (P_25_, P_75_)

Variables	BC group (n = 40)	1% Lido group (n = 40)	2% Lido group (n = 40)	Comparison among three groups, *P* value
Anesthesia time (min)	255.0 (210.0, 314.5)	255.0 (225.0, 315.0)	255.0 (225.0, 315.0)	0.814
Operation time (min)	190.0 (130.0, 243.8)	180.0 (152.5, 237.5)	180.0 (152.5, 237.5)	0.980
Sufentanil (μg)	25.0 (20.0, 25.0)	22.5 (20.0, 25.0)	25.0 (20.0, 25.0)	0.848
Remifentanil (μg)	1075.0 (762.5, 1575.0)	1000.0 (1000.0, 1500.0)	1000.0 (800.0, 1500.0)	0.489
Propofol (mg)	470.0 (350.0, 715.0)	575.0 (500.0, 687.5)	495.0 (400.0, 650.0)	0.105
Midazolam (mg)	1.0 (1.0, 1.0)	1.0 (1.0, 1.0)	1.0 (1.0, 1.0)	0.567
Dexmedetomidine(μg)	32.5 (0.0, 50.0)	0.0 (0.0, 38.8)	0.0 (0.0, 40.0)	0.073

*P* <0.05 indicates statistically significant difference. The use of various drugs was non-normally distributed, and the Kruskal–Wallis H test was used for inter-group comparisons.

### Primary parameters

RCSQ1–5 represented the following – RCSQ1: last night’s sleep depth, RCSQ2: last night’s rate of falling asleep, RCSQ3: last night’s number of awakenings, RCSQ4: last night’s rate of falling asleep again after awakening, and RCSQ5: last night’s quality of sleep, and the RCSQ score was the average of RCSQ1–5 scores. A higher score indicated better condition of the patient.

After removing the nasotracheal intubation and after the patient regained consciousness and could respond clearly according to instructions, we evaluated the patient for the presence of Horner’s syndrome, which is indicative of successful SGB. Notably, SGB was successful in all three patient groups. On the second day, the RCSQ was used to evaluate the sleep quality of the three patient groups on the first night after surgery.

**Figure 3.**
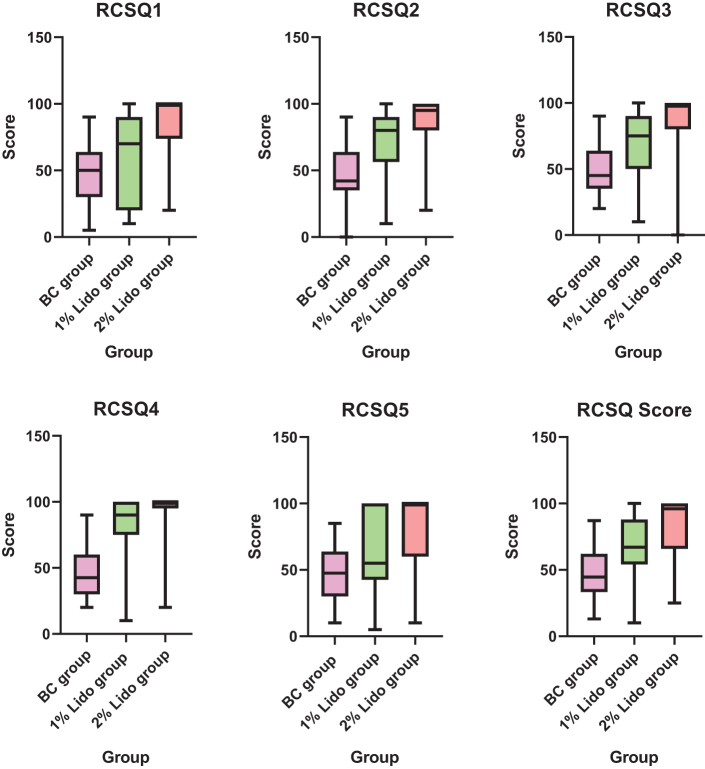
Comparison of RCSQ1, RCSQ2, RCSQ3, RCSQ4, RCSQ5, and the RCSQ score among the three groups.

As shown in Table 3, with statistically significant differences noted in RCSQ1, RCSQ2, RCSQ3, RCSQ4, RCSQ5, and the RCSQ score among the three patient groups. RCSQ1 and RCSQ5: These scores were statistically significantly higher in the 2% Lido group than in the BC group and 1% Lido group. RCSQ2, RCSQ3, and the RCSQ score: These were statistically significantly higher in both 1% and 2% Lido groups than in the BC group, and these were higher in the 2% Lido group than in the 1% Lido group. RCSQ4: This score was statistically significantly higher in both 1% and 2% Lido groups than in the BC group (as shown in Table 3). As shown in Fig. 3, among all categories, the BC group, 1% Lido group, and 2% Lido group showed a gradually increasing trend in scores (in this order), and the statistical differences between the values are detailed in Table 3.

**Table 3:** RCSQ scores of three groups of participants M (P_25_, P_75_)

Variables	BC group (n = 40)	1% Lido group (n = 40)	2% Lido group (n = 40)	Comparison among three groups, *P* value
RCSQ1	50.0 (30.0, 63.8)	70.0 (20.0, 90.0)	100.0 (73.8, 100.0)^a^^b^	**<0.0001**
RCSQ2	42.0 (35.0, 63.8)	80.0 (56.3, 90.0)^a^	95.0 (80.0, 100.0)^a^^b^	**<0.0001**
RCSQ3	45.0 (35.0, 63.8)	75.0 (50.0, 90.0)^a^	97.5 (80.0, 100.0)^a^^b^	**<0.0001**
RCSQ4	42.5 (30.0, 60.0)	90.0 (75.0, 100.0)^a^	100.0 (95.0, 100.0)^a^	**<0.0001**
RCSQ5	47.5 (30.0, 63.8)	55.0 (42.5, 100.0)	100.0 (60.0, 100.0)^a^^b^	**<0.0001**
RCSQ score	44.5 (33.3, 62.0)	67.0 (54.0, 87.9)^a^	96.0 (65.8, 100.0)^a^^b^	**<0.0001**

Bold: *P* value <0.05. *P* <0.05 indicates statistically significant difference. RCSQ1–5 and score were non-normally distributed, and the *Kruskal–Wallis H* test was used for inter-group comparisons, and the *LSD* text was used for pairwise comparisons.

^a^ There is a statistical difference compared with the BC group.

^b^ There is a statistical difference compared with the 1% Lido group.

### Secondary parameters

As shown in Table 4, at T1, T2, T3, and T4, there was a statistically significant difference in the gradual slowing of heart rate in the BC group, 1% Lido group, and 2% Lido group. At T1, the heart rate trend was as follows: BC group > 1% Lido group > 2% Lido group. There were statistically significant differences in all pairwise comparisons. At T2, the heart rate of the 2% Lido group was statistically significantly lower than that of the BC group and the 1% Lido group. At T3, the heart rate of the 2% Lido group was statistically significantly lower than that of the BC group. At T4, the heart rate of both 1% and 2% Lido groups was statistically significantly lower than that of the BC group. Other vital signs did not statistically significantly differ among the three groups (as shown in Table 4).

**Table 4:** Hemodynamic changes of three groups of participants ( $\overset{ˉ}{x}\pm s$)

Point of time	Variables	BC group (n = 40)	1% Lido group (n = 40)	2% Lido group (n = 40)	*F*-value	Comparison among three groups, *P* value
T0: Preoperative baseline value	MAP (mmHg)	90.0 ± 8.3	87.0 ± 7.6	87.2 ± 10.3	1.416	0.247
	HR (bpm)	81.0 ± 11.7	80.4 ± 10.2	78.9 ± 11.8	0.360	0.698
	SPO_2_ (%)	100.0 ± 0.3	100.0 ± 0.2	99.9 ± 0.3	0.114	0.893
	VAS	0.0 ± 0.0	0.0 ± 0.0	0.0 ± 0.0	/	**/**
T1: After extubation and awakening	MAP (mmHg)	82.5 ± 11.4	85.3 ± 11.8	88.3 ± 10.1	2.709	0.071
	HR (bpm)	97.3 ± 10.1	92.2 ± 13.6^a^	81.9 ± 14.4^a^,^b^	14.969	**<0.0001**
	SPO_2_ (%)	100.0 ± 0.2	99.9 ± 0.4	99.9 ± 0.4	1.417	0.247
	VAS	0.0 ± 0.0	0.0 ± 0.0	0.0 ± 0.0	/	**/**
T2: Immediately upon arrival at the AICU	MAP (mmHg)	89.1 ± 10.5	93.0 ± 11.9	94.7 ± 10.4	2.738	0.069
	HR (bpm)	97.0 ± 15.6	92.4 ± 14.1	82.0 ± 14.8^a^,^b^	10.636	**<0.0001**
	SPO_2_ (%)	99.6 ± 0.9	99.1 ± 1.2	99.4 ± 1.0	1.846	0.162
	VAS	0.9 ± 1.4	0.5 ± 1.0	0.5 ± 1.0	1.479	0.232
T3: One hour after arriving at the AICU	MAP (mmHg)	88.2 ± 10.9	89.0 ± 9.4	92.6 ± 12.0	1.909	0.153
	HR (bpm)	89.4 ± 14.8	85.4 ± 13.5	79.9 ± 14.8^a^	4.422	**0.014**
	SPO_2_ (%)	99.5 ± 1.0	99.3 ± 1.0	99.5 ± 0.8	0.938	0.394
	VAS	0.7 ± 1.2	0.5 ± 0.9	0.3 ± 0.8	1.604	0.206
T4: Two hours after arriving at the AICU	MAP (mmHg)	88.6 ± 9.8	88.7 ± 10.6	89.5 ± 9.8	0.106	0.900
	HR (bpm)	86.3 ± 14.0	79.5 ± 12.1^a^	78.8 ± 12.6^a^	4.141	**0.018**
	SPO_2_ (%)	99.5 ± 0.7	99.1 ± 0.9	99.4 ± 1.9	0.973	0.381
	VAS	0.3 ± 0.6	0.3 ± 0.7	0.0 ± 0.2	3.062	0.051

Bold: *P* value <0.05. *P* <0.05 indicates a statistically significant difference. *ANOVA* was used to compare MAP, HR, and SPO_2._ VAS scores were non-normally distributed, and the Kruskal–Wallis H test was used for inter-group comparisons. The *LSD* test was used for pairwise comparisons.

^a^ There is a statistical difference compared with the BC group.

^b^ There is a statistical difference compared with the 1% Lido group.

As shown in Table 5, there was no statistical difference among the three groups in terms of adverse reactions in patients within 2 h after surgery. As shown in Table 6, compared with the BC group, both 1% Lido group and 2% Lido group had significantly fewer supplemented doses of sufentanil, and there was no statistical difference in the number of supplemental midazolam *vs*. oxycodone *and* acetaminophen tablets among the three groups.

**Table 5:** Adverse reactions within 2 h after operation in three groups [case (%)]

Variables	BC group (n = 40)	1% Lido group (n = 40)	2% Lido group (n = 40)	*F*-value	Comparison among three groups, *P* value
Nausea and vomiting	1 (2.5)	1 (2.5)	1 (2.5)	0.000	1.000
Sore throat	32 (80)	24 (60)	30 (75)	4.268	0.118
Wound pain	12 (30)	13 (32.5)	8 (20)	1.755	0.416
Chills	0 (0)	1 (2.5)	1 (2.5)	1.017	0.601
Excessive oral secretions	0 (0)	1 (2.5)	1 (2.5)	1.017	0.601

*P* <0.05 indicates statistically significant difference. *χ ^2^* test was used to compare the differences among the three groups. If the expected count of more than 25% of cells was <5, the *Fisher’s exact* test was used.

**Table 6:** Number of postoperative sedative and analgesic drug supplements for three groups of participants [case (%)]

Variables	BC group (n = 40)	1% Lido group (n = 40)	2% Lido group (n = 40)	*F*-value	Comparison among three groups, *P* value
Midazolam	14 (35.0)	13 (32.5)	15 (37.5)	0.220	0.896
Sufentanil	15 (37.5)	8 (20.0)	6 (15.0)	6.093	**0.048**
Oxycodone and acetaminophen tablets	14 (35.0)	14 (35.0)	13 (32.5)	0.074	0.964

Bold: *P* value <0.05. *P* <0.05 indicates statistically significant difference. *χ ^2^* test was used to compare the differences among the three groups. If the expected count of more than 25% of cells was <5, the Fisher’s exact test was used.

## Discussion

Postoperative sleep disorders (POSD) often manifest as worsened sleep quality, sleep fragmentation, shortened sleep duration, and nightmares. Among the various perioperative complications, POSD are quite prevalent yet easily overlooked^[12]^. The incidence of POSD is reportedly 30–80%^[13–15]^. POSD are significantly associated with postoperative pain^[16]^, and perioperative sleep disorders have a detrimental effect on the short-term and long-term prognosis of patients^[17,18]^. The incidence of postoperative sleep disturbances is high in patients having undergone maxillofacial surgery because of tight bandaging of the head and face after the surgery, swollen and painful wounds, oral secretions, and a high level of discomfort, and the quality of sleep on the first postoperative night is particularly most significantly affected^[11]^.

The cervical sympathetic chain comprises the superior cervical ganglion, the middle cervical ganglion, and the inferior cervical ganglion. The subcervical ganglion fuses with the first thoracic ganglion to form the cervicothoracic ganglion, which is also known as the stellate ganglion^[19]^. Using blinding at C6, the main ganglion blocked is the middle cervical ganglion, whereas the stellate ganglion can be blocked only if the injection spreads to the level of C7–T1, and performing the procedure under ultrasound guidance improves the success rate of SGB^[19]^. SGB involves blocking the stellate ganglion nerve conduction by injecting local anesthetics, regulating the autonomic function of the area innervated by the stellate ganglion, dilating cerebral blood vessels, improving cerebral blood perfusion, reducing local reactive oxygen species, and synchronizing melatonin secretion rhythms^[7]^. Ultrasound guidance improves the success rate of SGB, effectively reducing the amount of local anesthetics administered^[20]^ and reducing complications as well^[21]^. Blind SGB, which is completely dependent on anatomical landmarks, is likely to damage the peripheral vascular nerves, and the quality of the block cannot be guaranteed, whereas ultrasound-guided SGB, which is easy and safe to perform, allows for direct visualization of the needle position and targeted diffusion of local anesthetics, thus improving the quality of the block and preventing serious complications^[6]^. Compared with puncture at the C7 transverse process, ultrasound-guided modified SGB at the C6 transverse process takes less time to perform and requires less adjustment of the puncture angle^[22]^.

Bilateral SGB can lead to bilateral recurrent laryngeal nerve paralysis and apnea^[23]^. Left SGB can reportedly impair left ventricular function and reduce the per-pulse output^[24]^. In a model of acute coronary artery occlusion, it was found that left SGB could not improve the balance of oxygen supply and demand and possibly increased the risk of myocardial ischemia; conversely, right SGB effectively modulates the balance of oxygen supply and demand^[25]^. Right SGB can increase the electrophysiological stability of ventricular myocardium^[26]^. In a hypertensive model, right SGB can reportedly reduce the apoptosis of myocardial cells and reverse the reconstruction of the left ventricle in spontaneously hypertensive rats *via* the regulation of apoptosis-related proteins^[27]^. Therefore, right SGB was chosen for this study.

Luo *et al*^[28]^ reported that the use of 5% bupivacaine for ultrasound-guided SGB was consistently effective in alleviating postoperative sleep disturbances in patients undergoing lumbar spine surgery. Liu *et al*^[21]^ confirmed that 4 mL of 0.375% ropivacaine for ultrasound-guided SGB could effectively improve sleep quality in patients with insomnia, with a lower overall risk than 6 mL or 8 mL ropivacaine block. Thus, 4 mL was chosen as the injection volume in the present study. SGB using procaine is also reportedly effective in relieving hot flashes and sleep disturbances in patients undergoing endocrine therapy after breast cancer surgery^[29]^. The effect of stellate ganglion block comes from the inhibition of sympathetic nervous system excitability, and the long-acting local anesthetic ropivacaine injection has a more lasting effect. However, considering the safety of patients and the short hospitalization time of maxillofacial surgery patients, this study chose two different concentrations of short local anesthetic lidocaine. The traditional local anesthetic lidocaine, which has a stable duration of action of 1.5–2 h and low cardiotoxicity, was chosen for this study, and two different concentrations of 1% and 2% were chosen for comparison in this study. In this study, the right SGB was performed under ultrasound guidance at the end of surgery and before removing the transnasal tracheal tube; within the time of lidocaine’s efficacy, it can reduce the heart rate 5-10 bpm, indicating a potential alleviation of the patient’s stress response at four different postoperative time points after extubation and effectively improve the quality of sleep of maxillofacial surgical patients for the first postoperative night, and the effect of 2% lidocaine (4 mL) was significantly better than that of 1% lidocaine (4 mL).

Our findings confirmed that compared to the blank control group, SGB in 1% and 2% lidocaine groups more effectively slowed the heart rate of the patients and reduced the stress response during the 2-h postoperative period, with 2% lidocaine being more effective than 1% lidocaine. Surgical and anesthetic manipulations also produce a strong stress response in the organism, which manifest as hyperexcitability of the hypothalamic–pituitary–adrenal axis and sympathetic–adrenomedullary axis^[30]^. Previous studies have shown that SGB reduces the post-traumatic stress response^[31]^ and decreases serum levels of norepinephrine and cortisol after laparoscopic surgery in patients with colorectal cancer^[30]^. Furthermore, it reportedly increases serum levels of melatonin in rats after sleep deprivation^[8]^. Luo *et al*^[28]^ demonstrated that SGB relieved postoperative sore throat. In patients undergoing laparoscopic resection of colorectal cancer, ultrasound-guided SGB was performed with 10 mL of 0.2% ropivacaine before the start of surgery, and the patients’ postoperative expiratory time was significantly shortened, plasma cortisol levels were reduced, and postoperative pain was effectively relieved^[32]^. The use of SGB using 6 mL of 0.5% ropivacaine after anesthesia induction made intraoperative hemodynamics more stable, led to lower inflammatory indices IL-6 and IL-10 in patients after laparoscopic resection of hepatocellular carcinoma, reduced postoperative inflammatory reaction, improved postoperative gastrointestinal digestion, and relieved postoperative inflammatory pain^[33]^. Preoperative SGB under ultrasound guidance can effectively improve postoperative sleep quality, reduce pain, and decrease the incidence of breast cancer-related lymphedema in breast cancer patients^[34]^. Furthermore, preoperative ultrasound-guided SGB can reduce the need for intraoperative isoproterenol, improve postoperative sleep and gastrointestinal function recovery, and consequently improve the quality of postoperative recovery in patients undergoing breast cancer surgery^[6]^. The results of basic research have confirmed that right SGB can improve spatial learning ability and minimize memory impairment in sleep-deprived rats, and its mechanism may be related to the reduction of hippocampal apoptosis and inflammation in sleep-deprived rats^[8]^. However, both concentrations of lidocaine for nerve block reduced the number of sufentanil doses on the first postoperative night, suggesting that they contributed to reducing postoperative pain.

The stellate ganglion is an important node in the sympathetic chain that regulates sympathetic activity in the head, neck and upper limbs. By SGB, sympathetic hyperexcitability can be reduced, anxiety and stress can be reduced, and sleep can be promoted. SGB is widely used to treat diseases related to autonomic nervous system dysfunction, such as insomnia, postoperative sleep disorders, anxiety disorders, etc. It has a particularly significant effect on patients with primary sleep disorders. It usually has a long-lasting effect, and patients can benefit in the long term after a single treatment. It usually takes several weeks or months before repeated treatment is needed.

Sphenopalatine Ganglion Block (SPGB) and SGB are both used in maxillofacial surgery, but there are obvious differences between the two in terms of anatomical location, mechanism of action, scope of application, and method of operation. SPGB reduces the transmission of pain signals by blocking the sympathetic, parasympathetic and sensory nerve fibers in and around the sphenopalatine ganglion, and it can also inhibit the pain reflex mediated by the trigeminal nerve to reduce facial pain^[35,36]^. The study showed that patients who received ultrasound-guided SPGB reduced the use of opioid drugs during and after surgery^[37]^.

SPGB mainly inhibits the activation of parasympathetic nerves, reduces the activity of pain receptors around intracranial and meningeal blood vessels, and decreases the release of neuroinflammatory mediators from sensory nerve fibers. SPGB is mainly used to treat headache related diseases such as migraine and cluster headache, indirectly improving sleep quality by improving headache symptoms, or indirectly improving sleep by improving rhinitis and nasal congestion symptoms. The persistence of the effect is relatively short and may require more frequent treatment to maintain the effect. High safety, but slight discomfort such as nasal irritation and temporary swallowing difficulties may occur^[38]^. The effect of SPGB on postoperative sleep in patients undergoing maxillofacial surgery could be our next target for our study.

### Limitations

This study has some limitations. (1) The age range of the patients was inadequately wide. (2) Polysomnography was not used after surgery, and a more subjective sleep questioning questionnaire was used. (3) The patients were not tested for changes in blood levels of the stress hormone cortisol on the first night after surgery. (4) The postoperative analgesic effect was poorly targeted, and it was not possible to specify exactly where and what kind of pain was present. (5) This study did not confirm that SGB provides relief from postoperative sore throat, which may be related to the small sample size and the short duration of lidocaine action. (6) The Limitations of the RCSQ relied on subjective patient reports; unsuitable for cognitively impaired or sedated individuals, assessed only short-term sleep (typically the previous night), did not address all sleep disorders (e.g., circadian rhythm disruptions, sleep apnea). (7) Due to the short hospitalization time of maxillofacial surgery patients, this study chose the short acting local anesthetic lidocaine and only followed up on the first night after surgery. If conditions permit, a 1 week follow-up can better observe the impact of stellate ganglion block on sleep. In future research, we intend to use polysomnography to evaluate changes in patients’ sleep rhythms, and with sufficient funding, we aim to monitor changes in patients’ cortisol levels.

## Conclusion

A single ultrasound-guided right SGB in maxillofacial surgery patients performed before removal of the tracheal tube at the end of surgery could effectively improve the quality of sleep on the first postoperative night according to RCSQ and reduce the 2-h postoperative stress response. In addition, administration of 4 mL of 2% lidocaine was superior to that of 4 mL of 1% lidocaine. However, both concentrations of lidocaine could reduce the number of supplemental sufentanil doses administered on the first night after surgery, making this a safe and effective method for improving postoperative sleep.

## Data Availability

The datasets generated during and/or analyzed during the current study are available from the corresponding author on reasonable request.

## Research registration unique identifying number (UIN)

The study was registered with the China Clinical Trials Registration Centre (http://www.chictr.org.cn) prior to participant recruitment (ChiCTR2300070330; principal investigator: Wang Ye; date of registration: 10 April 2023).
